# “New types of stories”: a narrative view of good nursing care of severely ill adult patients suffering an eating disorder

**DOI:** 10.1186/s40337-025-01345-4

**Published:** 2025-07-22

**Authors:** Berit Støre Brinchmann

**Affiliations:** 1 Regional Centre for Eating Disorders, Nordland Hospital, Bodø, 8076 Norway; 2 The Faculty of Nursing and Health Sciences, Nord University, Bodø, 8026 Norway

**Keywords:** Eating disorder, Narrative, Nursing care, Risk, Virtues.

## Abstract

**Background:**

This study is based on narratives about good nursing care from nurses with experience of working with adult patients with a severe eating disorder. Its aim is to explore and elaborate on nurses` stories. What do nurses highlight as being good nursing practice, and what can we learn from their accounts of good nursing care for people with a serious eating disorder?

**Methods:**

Riessman’s thematic narrative approach was chosen. Twelve nurses were interviewed individually and asked to reflect on stories from their nursing practice in which they had performed good nursing care. Four stories from these interviews were selected for this article. These four stories were analysed deductively, based on virtue ethics. The stories were worked on one at a time and then considered and analysed together toidentify differences and similarities.

**Results:**

The analysis shows how nurses, in addition to scientific knowledge and experience, apply virtues, professional judgement and phronetic knowledge, and take risks in the nursing of patients with a severe eating disorder.

**Conclusion:**

Stories of nurses are needed to complement the medical, psychological and diagnostic language used in the treatment of eating disorder patients and will further enrich both clinical practice and research.

**Supplementary Information:**

The online version contains supplementary material available at 10.1186/s40337-025-01345-4.

## Abstract (plain language summary)

This study is based on narratives about good nursing care from nurses with experience of working with adult patients with a severe eating disorder. Its aim is to explore and elaborate on nurses` stories. What do nurses highlight as being good nursing practice, and what can we learn from their accounts of good nursing care for people with a serious eating disorder? The results are discussed in the light of virtue ethics. The study sheds light on how nurses, in addition to scientific knowledge and experience, apply virtues, professional judgement and practical wisdom, and take risks in the nursing of patients with a severe eating disorder.

**Supplementary Information:**

The online version contains supplementary material available at 10.1186/s40337-025-01345-4.

## Background

Patients suffering a severe eating disorder (ED) will normally require intensive care and long-term follow up from highly trained and skilled nurses, as these are mental health conditions leading to not only significant suffering for those affected, but also serious somatic complications [1] and, in the worst case, death [2]. This nursing care includes, not least, activities around mealtimes and nutrition [3–5], but also monitoring, testing, feeding and, when needed, explicitly life-saving measures. These having been previously described elsewhere [5–7]. Nurses have an important role in the treatment and recovery of seriously ill inpatients with ED. Research into nurses’ experience is important for further development of knowledge about nursing for this patient group. Nurses’ stories can provide insight into nurses’ experiences. Stories often contain rich descriptions and can in this way put small but significant nuances into words and highlight knowledge that otherwise remains unarticulated.

Studies eliciting the views of ED patients indicate that other factors besides body, nutrition and food play a significant part in their recovery. A systematic review and qualitative meta-analysis found that psychological wellbeing and self-adaptability/resilience were fundamental to recovery from an ED [8]. All kinds of positive feelings and an optimistic outlook– feeling happy and cheerful, enjoying things, being calm and at peace as well as having good self-esteem and self-regard and warm, satisfying and trustful relationships with others– were all found to be helpful, as was a capacity for empathy, intimacy and openness. De Vos et al. [8] also emphasised the importance of personal growth, having a sense of direction, together with hopes, dreams and aspirations, feeling that one has a value for the wider society and that others value one’s efforts and activities. All of these were seen as being elements fundamental to regaining health. This corresponds to findings in a qualitative study investigating the reflections of young persons with a lived experience of anorexia nervosa (AN), and what they considered important for the recovery process [9]. Some of these were focusing on things that matter, finding a sense of mastery and leading a normal life where attention is on everything else than body and food, and being involved in important relationships and engaging with their future self. This message is underlined by a study by Rance et al. [10], revealing perspectives of adult women, who had been under treatment for AN. These women criticised the treatment regime for being overly focused on food and weight (p. 586) and pointed to genuine relationships with healthcare staff and therapists, and the possibility of being seen and addressed as whole persons, as being helpful during the recovery process. EDs are not about food, but about life. In a qualitative study by Smith et al., [11] interviews were conducted with 21 adult women with AN at a specialist inpatient ED unit. The findings suggest that patients experienced a process of change and adjustment in relation to levels of perceived personal control, attachment to the treatment environment and a sense of self-identity. Recovery was seen as regaining one’s character and personality, and was described as an arduous process, with contrast between physical restoration and AN recovery.

Conti et al. [12] make the point that, in addition to the usual medical/ psychological/ diagnostic language used in respect of ED patients, relational metaphors and a relational approach are needed to encompass the experience and complex feelings of these patients, and call for stories of this patient group:

We need a different kind of listening; an attention to metaphors that we do not know, plot lines we cannot follow and problems we have not fully recognized………. new types of stories will shift our understanding, and shape and change our research and therapy practices (12, p. 3 and p.5).

I haven’t found any articles based on nurses’ stories of this patient group and believe that stories of nurses also are needed to complement the medical, psychological and diagnostic language used in the treatment of ED patients.

The present study is based on narratives where nurses reflect on what they consider good nursing care for patients with a severe ED.

## Theoretical framework– virtue ethics

To further understand the nurses’ narratives of good nursing I present the theoretical framework of virtue ethics. “Ancient ethical theories are concerned with the agent’s life as a whole.……. concerned with character and choice, with practical reasoning and the role of the emotions” [13, p. 4]]. This calls for reflection on what a good life is and what amounts to “human flourishing”. In Aristotle’s Nicomachean Ethics [14] ethics are seen as virtue ethics– how can the moral agent best live their life? Aristotle argues that virtue is a necessary component of happiness, and that human flourishing requires that we are virtuous in accordance with reason [15]. In moral philosophy, virtue is a part of our character, informing our moral stance and our actions, enabling us to behave morally and well.

Aristotle [14] wrote:

.. but the virtues we get by first exercising them, as also happens in the case of the arts. we become just by doing just acts, temperate by doing temperate acts, brave by doing brave acts (NE II 1, 1103a32-b2) [14].

...to feel them at the right times, with reference to the right objects, towards the right people, with the right motive, and in the right way, is what is both intermediate and best, and this is characteristic of virtue (NE II 6 21b-23) [14].

Moral virtue must find the golden mean: neither too much nor too little [14].

Virtue ethics has had a renaissance in our time. In his book “After Virtue” [16], MacIntyre refers to Aristotle’s understanding of man, morality and virtue. He argues that modern society is characterised by individualism with life divided into segments. In pre-modern times, the self was perceived as a unit, built on one story about life (from birth to death). How we understand our own and others’ actions depends on how we construct our stories. These stories are also dependent on the tradition in which we stand; they are woven into the larger narratives that give life a unity. “…. because we all live out narratives in our lives and because we understand our lives in terms of the narratives that we live out……. the form of narrative is appropriate for understanding the actions of others” [16, p. 246]. Every life story is part of an interconnected bundle of stories. We are also part of other people’s stories, which makes us responsible: “I can only answer the question ‘What am I to do?’ If I can answer the prior question ‘Of what story or stories do I find myself a part?’ “ [16, p. 216]. Understanding the stories of the nurses in light of virtue ethics may give important clues to how nurses navigate the stories of their patients as well as their own attempts to do good for their patients and provide best possible nursing.

## Methods

### Aim

The aim of the study is to explore and elaborate on nurses` stories. What do nurses highlight as being good nursing practice, and what can we learn from their accounts of good nursing care for people with a serious ED? The results are discussed in the light of virtue ethics.

### Design

I conducted an exploratory, qualitative study, in which twelve nurses were interviewed. This article is based on a thematic narrative analysis [17, 18] of stories from these interviews. Storytelling offers understanding of human experience; the narratives situated in time, place and setting and constituting “essential meaning making structures” [17, 18]. I agree with Clandinin et al. [19] who argue that:

Stories and experiences require attention and care precisely because they are alive in people’s lives; they are carried forward from the rarified context of a research interview into the worlds of researchers, participants, and those with whom they interact. In this way the interview can not only shape stories on the page, but also the stories that become part of the world in which participants, researchers, and our research findings are embedded [19, p. 9].

### Study context

The nurses interviewed worked on an inpatient ward providing 24-hour care for adult patients with an ED (AN or bulimia) in a Norwegian psychiatric hospital. There is no consensus on the definition of severe EDs [20]. The ward in this study followed the diagnostic criteria for severe AN in line with the Diagnostic and Statistical Manual of Mental Disorders, Version Five (DSM-V): body mass index (BMI) < 15; Intentional caloric restriction resulting in weight loss; intense fear of gaining weight; and body image distortion (i.e. believing themselves to be extremely fat, when they are actually normal - or even underweight). In addition, the cognitive consequences of being severely underweight impact the capacity to consent.

The unit has eight beds, and approximately fourteen nursing positions, plus treatment staff. The patient group consisted of individuals receiving both voluntary and involuntary treatment. The patients were mainly women, most aged between eighteen and twenty-five, some in their thirties and forties. Some hospitalised for up to a year, some for a few weeks [7, p. 3].

### Recruitment and characteristics of participants

Information about the study was given during a staff meeting, and those wishing to participate contacted the author. The participating nurses comprised ten women and two men, between thirty-eight and seventy years old and with between two- and nineteen-years’ experience on the inpatient ward. Some were specialised in psychiatric nursing/mental health care, others in intensive care, diabetes, management, paediatrics, or public health. All had extensive work experience with other patient groups, within both somatic and mental health [7, p. 3].

### Data collection

After obtaining informed consent from the participants, I conducted twelve qualitative interviews at the hospital in 2022 using an interview guide (supplemental material). The main question was: Can you tell me as specifically as possible about a situation where you experienced that you provided good nursing care to a patient with a serious ED? Some of the respondents only answered the questions in general without specific examples. Other interviews contained more than one story. The interviews lasted between 30 and 70 min; 54 min on average. All were audio recorded and transcribed verbatim.

### Data analysis

Riessman’s thematic narrative approach was chosen [17, 18]. I read all the interviews carefully to identify nurses’ stories of good nursing care. The stories in a text often do not have clear-cut “borders,” and the researcher participates in the creation of stories, rather than “finding” them in the interviews by deciding what to present as stories [18, p. 21]. In line with Riessman [18], story in this study means “Bounded segment of interview text about an incident “(p. 75). The nurses’ stories were of varying length and quality, and some had a clearer focus than others. Several were about food, and the story about cheese was chosen because it had the most distinctive character. Four stories from four different interviews were selected for this article. This sample was not taken to be representative of the material as a whole but was found to be relevant to the research question ([18], p. 55; [21], p. 4). The selected stories show breadth and variety of nursing. These stories illustrated, through a variety of narratives and voices, differing aspects of good nursing care of those suffering a severe ED. What they all have in common is what I consider rich and interesting descriptions of holistic nursing for patients with a severe ED, and how the nurses use themselves in various ways in their encounters with the patient.

According to Riessman [18], prior theory might serve as a resource for interpretation of narratives (p. 73). Stories about the nurses’ use of themselves, as well as research eliciting the views of patients [8–11], such as emphasising the importance of personal growth, having a sense of direction, together with hopes, dreams and aspirations (of “the good life”), inspired the choice of virtue ethics as the theoretical framework for the article. The choice of virtue ethics was not decided beforehand.

Thematic narrative analysis focuses exclusively on the content of each story (what is said), keeping each story “intact”, studying each individually rather than theorising across the entire set [18, p. 53]…” stories must always be considered in context, for storytelling occurs at a historical moment with its circulating discourses and power relations” [18, p.8]. The stories were worked on one at a time, read and studied several times. All four stories were then considered and analysed together to identify differences and similarities.

The following questions, designed with a background in virtue ethics, were posed to the stories:

*What is at stake? How do nurses help patients with a severe ED to take back control of their lives? How do they meet the healthy part of the patient?*

*What do nurses do? What qualities and virtues are important when caring for patients with a severe ED?*

*What risks do nurses take when nursing patients with a severe ED?*

The questions asked to the stories are further elaborated in the results– and discussion sections.

### Ethical considerations

The study received approval from Norwegian Data Protection Services for Research (SIKT) (Ref. No. 947253). The nurses interviewed gave their informed consent, and the resulting data were anonymised and held in confidence.

## Results– four narratives from nurses

### Thea’s story– cheese

Thea is a psychiatric nurse. She is 43 years old and has extensive work experience from different psychiatric wards. She has worked for seven years at the inpatient ward for severe EDs.

Thea tells us:

*I was assigned to the team looking after a young woman suffering from AN*,* who had been here several times before: three times*,* I think. On those occasions she had been in voluntary treatment. This time*,* she was compulsorily hospitalised. She didn’t want that*,* but she was very*,* very sick. And it had come to my attention… because*,* you know*,* that…. I don’t really like to know too much before I actually meet people*,* I like to talk to them and form my own impression and not have so many preconceptions. But then I remembered that someone had told me that each time she had been with us previously*,* she had managed to not eat cheese: had not eaten cheese any of the times she had been here before*,* during all the time spent in treatment. And I thought… well*,* you might think that cheese has nothing to do with whether she gains weight or… she can gain weight on jam and on mackerel in tomato sauce. But then I thought*,* there must be something in your thoughts or your feelings making you so scared of this cheese. And cheese is just cheese. Perhaps you like it a lot. And then I recalled the first mealtime I had with this patient*,* that kind of one-to-one eating*,* and I put some cheese on a slice of bread. And the patient hyperventilated*,* cried*,* said that she wouldn’t*,* that she couldn’t. But we worked through it. She ate it. And later*,* during the treatment*,* it emerged that this was*,* in part*,* due to it being high in fat*,* a fear shared by many patients with AN. But it was also that she had such bad conscience in eating it*,* because she loved it so much*,* it was the best topping she knew.*

Thea’s account reaches a turning point when, towards the end of her treatment, she gets the patient to eat all sorts of cheese, and the patient subsequently sends Thea a greeting in which she confirms that she has continued to do so after the completion of her treatment. By approaching the patient gradually and patiently, Thea has helped her to re-engage with the food she likes the best, and takes most pleasure in.

Thea says:

*And then I thought that care is about helping patients to go back to something that had been normal before. If you love to eat a slice of bread with liver pâté but haven’t done it in five years*,* go back to it*,* right? So*,* we had this as a project whilst she was hospitalised. She had cheese on a slice of bread whenever she had the chance*,* every single day. Exposure therapy*,* right?*

*And so*,* it moved on to camembert*,* to brie to…. I got the kitchen to order in blue cheese*,* right*,* take back what you really like*,* what you really crave*,* and don’t let the disease decide. After her treatment was completed*,* I heard from another patient*,* recently admitted and who knew her*,* that she should give me her regards and say that she was no longer afraid of cheese.*

*So*,* my goodness*,* it is possible to think that you can live a long*,* long and good life without eating your favourite foods*,* but if you think about quality of life and that an ED is also a thought disorder*,* then avoiding doing something you really like is maintaining… keeping the ED alive.*

Interviewer: *But it went from her refusing completely and then during a conversation with you…?*

*Yes*,* a supportive conversation*,* like…. come on! Of course you’re going to eat cheese*,* you’re going to eat masses of cheese. You haven’t eaten it in months and years and now you must treat yourself and be kind to yourself. What’s the worst that can happen and what…. You’ll get the same support with every bite you know. A bit more*,* come on*,* we can do this.*

Interviewer: *Why do you suppose the other nurses hadn’t done it?*

*I don’t know. Maybe it had gone under the radar. It may be that one…because one can think that…. It’s more to do with nutrition*,* the most important is not what you eat*,* just that you eat and be nourished…yes. But no*,* it was quite fun. Fun to see how she coped afterwards*,* and how one could see her anxiety becoming less every time she ate cheese*,* so that there was less feeling of unpleasantness*,* and that one could work*,* in parallel*,* with the feelings that came after the mealtime. And lastly… that you can think about the treatment of EDs*,* if you think about good care and what is… yes*,* it’s great to think that good care is helping patients to gain weight. And then you can think that many people who have an ED are ambivalent about that weight gain. And sometimes it is the staff who*,* in a way*,* do most of the work. But then I think that*,* well*,* maybe that’s how it is in the beginning. But being able*,* in a way*,* to move forward together… when the treatment ends*,* the patients to be part of the team and actively involved in it and in their own treatment and… Otherwise*,* it will be our kilos they gain not theirs*,* and then there is the risk of relapse when they get home. Isn’t that right? You’ve heard those stories.*

In Thea’s account, we hear about a patient who had stopped eating cheese. This was the best sandwich filling she knew, but eating it gave her a bad conscience, both because it is high in calories but also just because she liked it so much. Thea says: “…. care is about helping patients to go back to something that had been normal before.” The aim was for the patient to be able to allow herself it, and to be kind and generous with herself.

Thea approached the patient with curiosity and patience. She was wise enough to be mindful that she was pressing the patient just enough– neither too much nor too little [14] to get her to start eating cheese again. What is important, “… is to move forward together– that the patients feel part of the team and are actively involved in their own treatment… otherwise it is our kilos they gain, not theirs. “ When trying to convince and persuade the patient, Thea could easily have been rejected (by the patient), so having to give up, in line with other nurses in the department who thought it had ‟…more to do with nutrition, the most important is not what you eat, just that you eat and be nourished…yes. ”

Theas’ story shows that Thea thinks a great deal about how to get her patient to see beyond the illness and how to strengthen hope for better times ahead. In the story, she points to the importance and value of the sensory and the immediate. It may be learning to take care of yourself and to treat yourself to good things, such as beginning to eat cheese again, if you love cheese. These results are consistent with research indicating what patients find important in the recovery of people with a serious ED [8–10], such as being calm and at peace and enjoying things [8], focusing on things that matter and finding a sense of mastery, leading a normal life [9] and having genuine relationships with healthcare staff [10].

### Emma’s story– From patient to student

Emma is 62 years old. She is a psychiatric nurse but has many years’ experience from different somatic units at the hospital. She has worked four years at the unit for EDs.

Emma tells us:

*I want to mention a story that came to my mind today. We worked with someone who has been committed for treatment*,* and who has been here before. And we– the team - have said to her that we want to support and help her*,* and we see that you are in a completely different place now than before. And that we will manage to get things done even though you are obliged to be here. It’s been quite special because she has begun studying at university. So yesterday I accompanied her the entire day. She is very underweight and for her to get permission [to study] someone from the staff has had to be with her from the start. And it’s fun to work in a way that lets you meet the patient in what is important to her. So just; “I’ve started studying*,* I want to carry on*,* “she blossoms*,* suddenly her voice is louder*,* eye contact*,* managing to put into words what was causing her such anxiety before we went to the university*,* but she knew we were going.*

Here, Emma talks about a patient who has been admitted several times, and who remains an involuntary patient with a very low bodyweight. What is special about this patient is that she has received permission to study at university, despite being committed for treatment. This represents a major turning point in her treatment– from being involuntarily committed for treatment, under a strict regime on the unit, to a great deal of freedom as a university student. This, naturally, involves a risk, and much can go wrong, despite her being chaperoned by a nurse. Nurses who know her see how she is changed by this. She is radiant and re-animated, has a sense of mastery and of resuming her life.

Emma continues:

*Yesterday it was me [who was with her at the university]*,* and the day before it was another little bit older person. And then she says that there are other people she knows of who attend*,* and she is so afraid that they will see that she is now a student and also see the staff who work here. So*,* we made a very specific plan where we agreed that we wouldn’t sit in the classroom*,* because that was completely unnatural: why were we there? We stood out like a sore thumb. So*,* we sat outside*,* kind of following her and…*.

Interviewer: *Be a bit discreet?*

*Yes*,* we were to be discreet. So*,* I said*,* “I won’t talk to you along the way. What are you going to do about lunch?” In this way*,* we have created a safe environment for her. “I’m in your team*,* I want to help you*,* what do I need to do so that it’s OK for you?” Because then*,* she managed to say that her anxiety was precisely because… Then*,* we did things that were a little outside of what… because we were supposed to be in the classroom. We found out that this just didn’t work. So*,* we took the taxi together*,* and then I walked behind her and saw where she went and then sat outside and read and thought blimey how out of place I am. I had to tell the others [laughter]. I thought*,* what am I doing*,* I must try not to look as though I’m sixty years old– but it’s obvious that I am sixty [laughter]. So*,* I sat outside and read and tried to hide a bit. And I saw her leave. Yes*,* I did the same as her*,* so when she went to the toilet when there was a break - because we had the times - she told me where they were supposed to be when*,* and that they would be moving on in groups later - I saw her go to the toilet*,* and went out so that I wasn’t there when the others had a break. And went back in when they had…. and so*,* it was keeping an eye on the clock and being kind of discreet. Because she said she had stayed sitting in the classroom*,* because she didn’t want to go out to where I was. And then there was lunch*,* because she was going to eat there. It was the first time. Yes*,* how were we going to do that? No*,* there wasn’t any canteen; a lot of social anxiety and…and she said then that she was going to sit down*,* and I said; “Then I’ll just sit there discreetly too.” She had a packed lunch with her*,* and we had agreed that she would have a glass of milk which she was to drink later*,* before she went back. And then I said*,* “You can have juice”. No*,* she wouldn’t have that. “No*,* ok*,* then I’ll take the juice.” So*,* she sat herself down– a good distance away from me but I could observe her. So*,* I sat there and read*,* looked at my phone a little and at others but not at her other than a sideways glance. She ate the three slices of bread*,* but someone I had studied with years before came by. “Hi*,* have you started here?” “No…” and I didn’t go further into that. And so*,* she moved. And I stood there and chatted a little with the woman I had studied with. And with that the patient went back to the classroom*,* and I thought that she had dealt well with everything we had agreed. So*,* I just went outside and sat down in the fresh air. And class was supposed to finish at 2pm*,* so I was a bit on and off*,* but we were actually there until it was almost 3pm. And by then I was exhausted from the hiding. But then I said*,* “How did it go*,* did we manage our project?” “Yes.” So*,* things like that are kind of fun.*

In Emma’s story we see that the hope for a good life, and the sense of flourishing can be strengthened when you finally feel able to, and are given the opportunity to, study at university. “She is blossoming, speaks less quietly and makes eye contact”.

Aristotle argues that virtue is a necessary component of happiness, and that human flourishing requires that we are virtuous in accordance with reason [15]. Nursing covers the full span of human activities: coping with life, encountering different situations and dealing with them. Nurses use their personal qualities, but also moral virtues as defined by Aristotle. In Emma’s account, we saw how she improvised, used her experience, imagination and wisdom to help and support her patient at the university, and in different ways, in taking back her life to something more like it had been before the ED took over. Emma contributed to the self-development of her patient. She had confidence and courage to tolerate unease, not be faint-hearted. Emma displayed both courage and discretion while accompanying the patient to the university. She used her imagination and improvised, sitting and reading and hiding a little so that no one would become aware that she was there to follow up and observe the patient. Not least, she showed confidence in the patient and that she trusted her to manage on her own and that she would eat sufficient, even unsupervised. Virtue comes from encountering new situations and crises in this way: neither too much nor too little anxiety [14]. This is how the possibility of change and growth, essential if the patient is to escape their stuck and self-maintaining patterns, is created.

Emma also took a risk when she granted the patient more freedom than agreed at the university. She should really have joined her in the study room, but elected to sit outside in the hallway, so as not to embarrass her. Here Emma decided to be a little disobedient, ‟breaking the rules”, which naturally involves a risk. “We did things that were a little outside…”. On this occasion it went well, and the patient ate as required of her. She did not betray the trust placed in her. But Emma could not have known in advance how it would turn out, and that she would succeed.

Nursing is not about following a recipe. It calls for nurses able to make assessments as to what is professionally sound and caring nursing in any given situation. There is always a risk. Taking this risk seriously, without seeing it as inevitable, involves embracing nursing’s openness and unpredictability, facing an event that may or may not happen, recognising nursing’s limited power and seeing that nursing always needs the logic of phronesis because without the risk, judgement disappears from nursing [22, p.140].

### Oliver’s story– the passport photo

Oliver is 38 years old. He is a psychiatric nurse and has worked on different psychiatric wards, as well as eight years on the ED unit.

Oliver says:

*I think a lot about how I can do it*,* how I can reach out to the patients to make their stay here better*,* make it more tolerable. Just yesterday I was thinking about the patient I have now*,* who has a BMI of 10. How best can I prepare myself for the fact that this is a patient who may well be here for the rest of the year. I’m going to struggle with how lovely and fine she is*,* but at the same time how tragically small the chance is of her having a normal life. I’ll set this aside somewhat and talk to her each day as if this is bound to go well. But then how do I convey to the therapist what I’m thinking*,* how do I get across to them that we may have to break some rules for the patient to feel heard and understood*,* and how should I talk to the others about this*,* without it being about me or my needs*,* but that it is… that I am advocating all the way for this patient? And then*,* I have to work on myself all the time.*

*Today*,* I did something very special. The patient has a passport with her*,* and in the passport*,* she is… she says that she is 17*,* but I think that in the passport photo she is more like 15 or 16. And then she weighed 60 kg. That was before she came to us for the first time*,* and that was around a year ago. So*,* to put it this way; if she was 16 then… she will be 21 this summer. Then in those four years… and now she is 35 kg. And there is a completely different face [in the passport photo]*,* a completely different girl. And that girl is beautiful*,* an unbelievably pretty girl who has had a tough childhood. I don’t know everything*,* but now there are several diagnoses… But what I elected to do with this passport photo was to first*,* in a way*,* to approach her….“This*,* after all*,* is you. Look at you*,* you’re not just the one lying in bed here not eating. This here is you*,* look!” Making her curious about herself…was what I imagined*,* make her curious about who she is and accept who she is really*,* or was. Because now she is someone who has armpits that go right up into her shoulders. A BMI of 10 is among the most ill you are ever likely to see. But I said to her; “This photo*,* this passport*,* can I take it with me to the meeting today? I want them to see who you were and who you are. How you look.” “Yes*,* just do it.” “Yes*,* but are you sure? I just want… I just want to at least show you to your therapist*,* that your therapist and consultant see you”. Yes*,* yes*,* there was no problem; “You can just do it.” Kind of proud*,* I don’t know. And I took the opportunity; I took it with me and showed it to them. And I wasn’t that bothered about them seeing it. What I was interested in was whether it could create some interest in her*,* in who she was.*

*It could easily have been that this didn’t meet its mark. It could well have been that this seemed very strange to her*,* and that it was simply politeness that led her to say yes. So*,* I do take chances. Nursing is an art of one or another kind; the art of being genuine.*

Oliver reflects on how he could reach a very sick and underweight patient, and we hear about the way he improvised and used his imagination in this approach by showing her, and talking about, her passport photo, taken before she became so ill. He said that, in this way, he hoped to spark an interest in her, in who she was, and he asked if he could show the photo to those treating her. “And I wasn’t that bothered about them seeing it. What I was interested in was whether it could create some interest in her, in who she was”….

Oliver is explicitly clear that he took a risk in showing the patient her passport photo from before she became ill: “It could easily have been that this didn’t meet its mark. It could well have been that this seemed very strange to her, and that it was simply politeness that led her to say yes. So, I do take chances.”

He refers to nursing as being, in some way, an art - the art of being genuine. What does it mean to be genuine? It can mean being authentic, whole, sincere, or original; to use oneself. In his story, Oliver uses the passport photo to get the patient to take an interest in herself; other nurses might perhaps use themselves and their creativity in other ways to reach out to the patient.

Aristotle mentions ‘art’ as being an important part of practice [14].

An art in the Aristotelian sense of a practical activity [is] that [ it] has a purpose and that [it] is located in what Aristotle calls the domain of the variable. This is a domain where there are actions and possible consequences, but where there is never certainty about what may happen. You can say the artistry is to get closer and closer to the dynamics of that so that you can bring more experience to the practice, more resources, more ways of doing, but each time, you meet a new situation and the artistry is to figure out what needs to be done in that situation [23, p. 105].

### Molly’s story– suitably strict

Molly is 61 years old. She is an intensive care nurse and has worked on both somatic and psychiatric hospital wards. She has 16 years’ work experience from the inpatient ward for EDs.

Molly says:

*The episode I’m going to speak about is so fresh and recent that there’s no difficulty in recalling it. I can say that the headline for the whole thing is ‘Care’*,* in the sense of trying to see the patient*,* and of getting into the patient’s world of experience*,* and trying to act based on that. And these patients often challenge us in different ways*,* and it is very easy for common perceptions about the patient to spread among the staff group: they are extremely contagious. So*,* there is something about being able to resist this and being able to keep a cool head in everything that happens*,* with the patient who challenges you*,* but at the same time distinguishing what is… if you have been coloured by other things.*

*We received a patient who had been with us before and who was thought of as a so-called ‘difficult’ patient*,* demanding and something of a ‘hot potato’. We didn’t quite know how to act: should we keep strictly to the line or make lots of allowances. The times she has been with us previously*,* we have tried to be quite strict*,* keeping her well boundaried. That’s what we call it when we’re a bit strict. So*,* when she came and found out that I was going to be part of her team*,* she said that she really didn’t want to have me in the team. “Okay*,* but then you have to tell me what makes you say that?” Then she said that; “No*,* it’s just that I think you’re… can be so strict and can seem a little bit cold and a little bit… No*,* I don’t want you in my team.”*

Molly speaks of a patient whom she is familiar from an earlier hospital stay. The account contains the patient’s story but also nurse Molly’s on her contact with this patient, both now and earlier. The patient expresses her wish not to have Molly in her team, as she finds her to be too strict. And Molly is self-critical, reflects over this, admitting that, on occasions, she may have been irritated and overly strict.

Molly asks the patient:

*“What do you really need from me? If I’m going to continue to be in your team*,* what do you need from me if I’m going to try to help you*,* on the one hand*,* with your ED*,* but on the other in a way that you can be comfortable with?” “No*,* I don’t know.” She was a person of few words*,* so it’s not so easy for her to express herself. But*,* on this particular day*,* it was just as if she… as we spoke a lot came up*,* she became very verbal during that time*,* the three quarters of an hour we talked. Then I asked her; “Shall we agree to try? I’m on your team*,* and if we can’t make it work*,* we’ll call it a day. But I want us to try to see what we can achieve if we just manage to have a good dialogue.” So*,* then we agreed that we would.*

*And what I learned from that is that we must never stop listening to what the patients actually say. And there was a lot of noise around her*,* because she says ‘no’… she wants attention*,* and ‘yes’*,* now she has everything the way she wants it. This is typical*,* and now she acts out this way and that*,* and now she acts out this and that*,* right? And it is the question of balance in how to move in that terrain. It’s not entirely easy*,* but at least I would say that the process she was in - now I must add that she is still struggling with her stuff - but we eventually developed a pretty good relationship.*

*That which she considered strict… well*,* it can certainly be the case that I was a little unnecessarily strict*,* it may be that there was countertransference and that I was somewhat irritated. And there’s something in acknowledging that yes*,* it was actually… you actually were. But what she meant by me being strict was… what she said specifically was that I didn’t give her that extra pat on the back when we had to force feed her*,* that I was perhaps a bit*,* so to speak*,* technical. And I don’t believe that I have been insensitive or done things in an unkind way. But*,* nonetheless*,* she has had the experience that I was not sufficiently empathetic in that situation*,* especially during the force feeding. And the idea behind that? It had been communicated to us in the ward that we had to be a little careful not to be too warm and loving in force feeding situations*,* because that can*,* in some way*,* trigger a desire for empathy in those situations*,* and maybe lead to her not eating*,* going instead all the way to being force fed*,* because then she knows she will get a little extra attention. It can be very unfortunate to give too much. But you shouldn’t either… it’s a balance*,* and you shouldn’t be rude or insensitive either.*

*And there was a situation that became very difficult*,* when she needed a special exception. And there was a good deal of agreement in the ward that no*,* she shouldn’t get that*,* and where I thought that now we have to keep a cool head. It is absolutely right that we should not be facilitatory in all situations*,* without any limits. But in this case*,* it was actually the right thing to do. And I did it because her mother had called the ward and asked to speak to me. Then she said I have asked to speak to you because she*,* the patient*,* says you are the only one who will listen to me.*

*There was talk of her being allowed to use a different toilet than the one in there because there was something making it difficult for her to use it. And the mother said that this has been a problem since she was little. She is not able to go to the toilet if there is any chance of people outside hearing her. So*,* she’s just like that. While others in the ward thought no*,* that was to go give in to her… like all the requests and….*

Molly, who the patient found to be too strict, could easily have risked being rejected. It was by no means certain the patient would agree to the arrangement that was reached between them: “Shall we agree to try?” I’m on your team, and if we can’t make it work, we’ll call it a day.” In this situation, the patient could well have insisted on having a different nurse, one she found less strict. Towards the end of Molly’s account, a significant change is reached. From having seen Molly as being too strict, and not wanting her in her team, it is Molly the patient asks her mother to ring, so that she be allowed to use a toilet matching her needs. Now, it is the other nurses who are experienced by this patient as being strict. By engaging the patient in a dialogue, making an agreement with her and reflecting over her own practice, being self-critical, we see that Molly gradually becomes trusted by the patient, whilst– at the same time– demonstrating that good nursing, in relation to this patient, involves being sufficiently strict.

According to Aristotle moral virtue must find the golden middle way between extremes, not too much, but not too little [14].

……to feel them at the right times, with reference to the right objects, towards the right people, with the right motive, and in the right way, is what is both intermediate and best, and this is characteristic of virtue (NE II 6 21b-23) [14].

One might not normally think of strictness as a virtue, rather as a vice. However, with patients with a serious ED, being strict can, of necessity, be a virtue. Molly speaks about a patient who had been hospitalised several times previously and who said that she did not want Molly in her nursing team as she thought that Molly “could be…. strict and could seem a little cold.” Molly listened to the patient, reached an agreement with her that they could try having her in the patient’s team. At the same time, she reflected over her earlier experience of the patient, accepting that she had maybe sometime been irritated with her, and perhaps too strict. At the same time, there is agreement that this patient group needs firm boundaries [24].

In Molly’s account, we see that ‘being suitably strict’ has become an integral part of Molly`s phronetic knowledge or practical wisdom, that is wisdom enabling one to know what is fitting in a particular situation [14]. This includes consideration, judgement and experience. Molly may be seen as a model by other nurses, using herself as an instrument and being seen to be genuine. This authenticity leads to her being experienced as capable, experienced and wise. Genuineness is individual, whilst nurses might be tempted to copy Moly’s ‘suitable strictness’, copying seldom offers a good result. They must find their own authenticity.

Molly says that….” nurses must never stop listening to what the patients actually say…. And it is the question of balance in how to move in that terrain”.

## Discussion

The aim of this article was to explore and elaborate on nurses` stories. What do nurses highlight as being good nursing practice, and what can we learn from their accounts of good nursing care for people with a serious ED?

The stories were taken from four nurses who describe the nursing care of four different patients. There are considerable differences between the stories, and between the nursing and the patients described. Some patients are very ill, like Oliver’s patient with a BMI of 10. Other patients are healthier, so they can eat cheese or study at university. Common to all the patients is suffering from a serious ED, which means that they must be hospitalised, and be dependent on good nursing care.

We have seen that the nurses are concerned with how they in various ways can get the patients to take back control of their lives, by resuming something they liked before (Thea (Cheese)), trying new things, (Emma (From patient to student)), or becoming curious about themselves again (Oliver (The passport photo)). The analysis shows that all nurses use different virtues such as patience, persistence, imagination, creativity, improvisation, curiosity, and strictness, as well as using themselves in encountering their patients. “Nursing is the art of being genuine.” (Oliver). MacIntyre [16] refers to virtues as something that goes beyond what we usually think of as professional skills. Virtues can be manifested in very different situations, where one cannot expect virtues to be effective in the same way as professional skills (p. 238). “The virtues therefore are to be understood as those dispositions which will not only sustain practices and enable us to achieve the goods internal to practices, but which will also sustain us in the relevant kind of quest for the good….” (p. 254).

We have seen that all nurses take risks in different ways, when they try to reach out to and help patients with a serious ED to get better or wards a better life.

The accounts from the nurses show that they take risks based on experience and forethought, improvise and use their imagination. Nursing involves uncertainty. This will always involve there being some form of risk, because one can never know in advance how the patient will react, despite the good intentions of the nurse. The nurses’ actions could entail risks. Oliver (The passport photo) is expressly clear that he took a risk by showing the patient her passport photo from before she became ill: “It could easily have been that this didn’t meet its mark… So, I do take chances.” Thea (Cheese), who tried to convince and persuade the patient to eat cheese could have been rejected (by the patient), so having to give up. Molly (Suitably strict), whom the patient found to be too strict, had risked being rejected. In that situation, the patient could well have insisted on having a different nurse, one she found less strict.

Nursing always involves a risk, not that nurses fail because inadequately qualified, not through lack of scientific evidence, but rather because nursing is not about filling a bucket but about lighting a fire [22, p.1].

There is a risk in [nursing] because it is about human beings meeting each other and trying to figure out what it means to live their lives well.……as [nurses] we are always risking ourselves because we are interfering in the lives of other people which, in a very fundamental sense, is not something they asked for. So, there is a risk in that too [23, p.102].

In Emma’s story (From patient to student), we see the nurse deciding to be a little disobedient, ‟breaking the rules”, which naturally involves a risk. Emma took a big risk, giving her patient more freedom than agreed at the university. “We did things that were a little outside.”

Biesta [22] writes:

[Nursing] always deals with living “material,” that is, with human subjects, not with inanimate objects. The desire to make [nursing] strong, secure, predictable, and risk-free is an attempt to forget that at the end… [nursing] should aim at making itself dispensable– no [nurse] wants their [patients] to remain eternal [patients]– which means that [nursing] necessarily needs to have an orientation toward the [recovery], freedom, and independence of those being[nursed] [22, p. 2].

The stories show that the nurses are committed, they genuinely care and take responsibility. MacIntyre [16] says that every life’s stories are part of an interconnected bundle of stories. Patients` stories are part of nurses’ stories. This is particularly evident in Molly’s account (Suitably strict) where Molly talked about the patient who did not want her on her team, because she had experienced Molly as too strict, and Molly then took self-criticism, and reflected on it, and admitted that she might have been too strict. Oliver (The passport photo) also shows how his story is influenced by the patient’s story:

*I think a lot about how I can do it*,* how I can reach out to the patients to make their stay here better*,* make it more tolerable. Just yesterday I was thinking about the patient I have now*,*…. How best can I prepare myself for the fact that this is a patient who may well be here for the rest of the year…. and how tragically small the chance is of her having a normal life……*,* I have to work on myself all the time.*

According to MacIntyre [16] being part of other people’s stories, makes us responsible. The basic elements of all stories are to ask what I did and why, and what you did and why. We must not only seek the good as an individual, but also for what is good for others.

Realising that nurses’ stories are part of the patients` stories requires responsibility as well as actions. The nurses must use themselves and be willing to take risks. This seem to be an important part of professional judgement and phronetic knowledge in the nursing of these patients, and might be considered a meta-narrative of nursing patients with a severe ED.

### Methodological reflections

The author, Berit, is a 67-yearold trained nurse with a PhD. I have no clinical experience with ED patients but work as a researcher on a hospital ward for EDs, and as a university professor. In addition to nursing and EDs, my research interests include ethics and ethical challenges. Although I do not have direct experience with EDs, I have experienced serious mental illness in family and among friends and have seen how invasive it can be and how important it is to have skilled, dedicated and wise professional helpers. According to MacIntyre [16], we are part of each other’s stories. The patients’ stories are part of the nurses’ stories. The nurses’ stories are part of the researcher’s stories, and this article is based on my stories of the nurses’ stories. Riessman [18] writes:

By our interviewing and transcription practices, we play a major part in constituting the narrative data that we then analyze. Through our presence, and by listening and questioning in particular ways, we critically shape the stories participants choose to tell (p. 50).

The choice of virtue ethics as a theoretical frame of reference is also clearly influenced by my preconceptions and interest in virtue ethics.

I have not found any other studies that are based on nurses’ stories about good nursing care for patients with a severe ED. The stories show that small nuances can be significant for the patient and the nurse and can help to bring out rich descriptions of a practice that is often inarticulate. Nurses and researchers can learn from stories. Stories can also move us emotionally [18, p. 9], and inspire practitioners and researchers to tell new stories. In this way, stories can be important for both practice and research, and mobilise to action [18, p. 9]. It may be a limitation that the stories in this study are taken from nurses from the same department at a single hospital. Later studies should include nurses from different hospitals and also explore possible differences in stories from nurses with different work experience. Later research may also include contrasting and heterogeneous narratives, that could help understand complex care.

## Conclusion

This study shows how nurses, in addition to scientific knowledge and experience, apply virtues, professional judgement and phronetic knowledge in the nursing of patients with a severe ED. The nurses tell us that they use patience, persistence, imagination, creativity, improvisation, curiosity, and strictness, as well as using themselves in encountering their patients. “Nursing is the art of being genuine.” Not least do the nurses take risk when they try to encourage and help patients with a severe ED to improve and take back control of their lives. Realising that the nurse`s story is part of the patient`s story, requires responsibility and action. The stories of nurses are needed to complement the medical, psychological and diagnostic language used in the treatment of ED patients and will further enrich both clinical practice and research.

## Electronic supplementary material

Below is the link to the electronic supplementary material.

Supplementary Material 1

## Data Availability

No datasets were generated or analysed during the current study.

## Abbreviations

AN: Anorexia nervosa
BMI: Body mass index
ED: Eating disorder
