# Supplementary material for: “New types of stories”: a narrative view of good nursing care of severely ill adult patients suffering an eating disorder
Source: J Eat Disord. 2025 Jul 22;13:150. doi: 10.1186/s40337-025-01345-4 (PMC12285101; doi:10.1186/s40337-025-01345-4)
Supplement: Supplementary file 1 — Supplementary Material 1 [file 40337_2025_1345_MOESM1_ESM.docx]

Interview guide: (individual interview: nurses – adult patients` ward

How old are you?

What education do you have?

What work experience do you have?

How long have you been working with patients with eating disorders?

Tell about your workplace

Tell about some experiences that have made an impression on you as a nurse with eating disorders

Talk about a situation where you felt that you were practising good nursing/care – preferably as specifically as possible

Share a situation in which you experienced an ethical dilemma

What are your thoughts on rules and discretion?

What thoughts do you have about the use of coercion?

How have you experienced cooperation with relatives?

What is particularly rewarding about working with this patient group?

What is particularly difficult/challenging about working with this patient group?

Why does this patient group need nursing?

What do you think the patient/relatives think is good nursing?

Do you have something else important to convey, that others can learn something from?

What would you ask if you were the researcher?

I have now asked you as a nurse what you think is good nursing/care for people with

eating disorders. What do you think a patient/family member would have answered to the same question?

Is there anything important we haven't talked about?
